# Microstructure Evolution and Formation of Gradient Structures in Single Crystal Nickel-Based Superalloy by Surface Mechanical Creep-Feed Grinding Treatment

**DOI:** 10.3390/ma16010321

**Published:** 2022-12-29

**Authors:** Qing Miao, Wenfeng Ding, Weijie Kuang, Bijin Zhou, Ting Hao, Chenwei Dai, Zhen Yin

**Affiliations:** 1 College of Mechanical Engineering, Suzhou University of Science and Technology, Suzhou 215009, China; 2 National Key Laboratory of Science and Technology on Helicopter Transmission, Nanjing University of Aeronautics and Astronautics, Nanjing 210016, China

**Keywords:** gradient structures, surface mechanical creep-feed grinding treatment, hardness, single crystal nickel-based superalloy

## Abstract

Gradient structures have been created in single crystal nickel-based superalloys (SX alloys) via surface mechanical creep-feed grinding treatment (SMCGT). It has been found that these gradient structures are mainly composed of nano-sized grains, sub-micron-sized grains, dislocation structures, and the matrix material of single crystals along the depth from the treated surface. In addition, the evolution of such structures is found to be dominated by the dislocation movements which run through both γ channels and γ’ precipitates, subdividing the two types of microstructures into various dislocation structures, and eventually introducing the refined grains into the surface layer. Furthermore, the evolution process of gradient structures primarily originates from the mechanical effect between abrasive grits and workpiece material, owing to the large grinding force (up to 529 N) and low grinding temperature (less than 150 °C) during the unique creep-feed grinding treatment in the present investigation. Due to the typical grain refinement, the hardness of the nanostructures exhibits the largest value of around 10 GPa in the surface layer, approximately 26% higher than that of the matrix material. This study further enhances the understanding of the microstructure–property relationship of SX alloys subjected to creep-feed grinding treatment and contributes to achievement of high-performance components.

## 1. Introduction

Nickel-based superalloys are of increasing importance in the aero-engine and nuclear fields due to the combination of superior mechanical strength and outstanding corrosion resistance [1,2]. However, owing to extreme working environments (i.e., high temperature and high pressure) in practical applications, fatigue failure of nickel-based superalloy components induced by crack faults and fractures often occurs, thereby threatening the safety of humans [3,4]. To improve the service performance of nickel-based superalloys, several effective surface strengthening techniques have been developed, including high-pressure torsion (HPT) [5], laser shock processing (LSP) [6], surface mechanical attrition treatment (SMAT) [7], surface mechanical rolling treatment (SMRT) [8], and surface mechanical grinding treatment (SMGT) [9], etc. In particular, SMGT not only serves as a common machining method, but it also produces an extremely large strain and strain rate at the machined surface of nickel-based superalloys through strong thermo-mechanical interactions between abrasive grits and the workpiece. This process can create severe plastic deformation (SPD) layers, refine coarse grains, introduce deep compressive residual stresses, and improve surface quality and mechanical properties [10].

It is well known that the microstructure of superalloys dominates its mechanical behaviors. Thus, extensive endeavors have been made to enhance the understanding and the coordinated controlling of the microstructure performance of such alloys by means of various surface strengthening methods. Ortiz et al. [11] studied the nanocrystalline surface layer of a C-2000 nickel-based alloy induced by SMAT. The average grain size was refined from 50 µm to 12 nm. Large residual compressive stresses were also produced in the surface layer. Liu et al. [12] used SMGT to produce a novel nano-laminated structure with low-angle boundaries in pure nickel, whose size was about 20 nm on average. Further, experiments have demonstrated that this typical structure exhibited high hardness (around 6.4 GPa) and great thermal stability. Using a similar method, Ding et al. [13] introduced gradient structures (consisting of a surface nano-laminated layer and a deformation twinned layer) into a C-22HS nickel-based alloy via SMGT. It was found that the grain sizes were even below 30 nm at a 2 µm depth from the treated surface, and the yield strength of such gradient structures were around four times bigger than that of the matrix material. Lu [14] reported that the gradient nanograined surface layer of engineered alloy could increase the fatigue limit by 100% because the fatigue crack initiation was suppressed by the hard and ductile nanograined skin, and that crack propagation can be arrested by the soft coarse-grained interior. In addition, Bagui et al. [15] demonstrated that the microstructure evolution of γ’ precipitates had significant effects on the deformation behavior of an Inconel 617 superalloy at high temperatures (650–800 °C). The above literature indicates that both the refined grains and the depth-dependent gradient structures are beneficial in enhancing the service performance of nickel-based superalloys, for instance, in the creep property [16], the wear resistance [17], and the corrosion resistance [18].

For single crystal nickel-based superalloys, the γ and γ’ phases co-exist together with multi-elements in solid solution structures, and they influence each other during SMGT. Recently, some of the literature has also reported grinding-induced changes in microstructures and the mechanical properties of the surface layer at a macro/micro-scale [19,20,21]. However, some important issues, such as the structure evolution at the nano scale and its effect on mechanical properties and the interactions between γ and γ’ phases during SMGT, are still pending. Particularly, creep-feed grinding, one of the high-efficiency machining methods used to obtain the high surface quality of SX alloys, has been commonly employed in the aircraft industry. Nevertheless, because of the sensitivity of practical applications of SX alloys, the conservative process parameters are used usually to minimize the changes in workpiece material microstructures and the related properties, thus reducing the surface strengthening effect and even limiting improvements in machining efficiency. In other words, high-performance SX alloy components treated by creep-feed grinding cannot be achieved unless the microstructural changes and the underlying mechanism are revealed. Therefore, it is very urgent to figure out the microstructure evolution of SX alloys induced by surface mechanical creep-feed grinding. However, such aspects are studied insufficiently.

To this end, the present work is concerned with the gradient nano–microstructures in the surface layer of single crystal nickel-based superalloys, induced by surface mechanical creep-feed grinding treatment. The arrangement of the present study was as follows: firstly, the surface layer of the SX alloy after SMCGT was examined by scanning electron microscopy (SEM); secondly, the microstructure evolution and the interactions between the two phases were further characterized by transmission electron microscopy (TEM) with high magnification; thirdly, the primary mechanism dominating the gradient structures in the SX alloy during SMCGT was discussed from the point of view of dislocation activities; finally, the effect of the gradient structure on the cross-sectional hardness of the SMCGT sample was evaluated.

## 2. Materials and Methods

**Figure 1:**
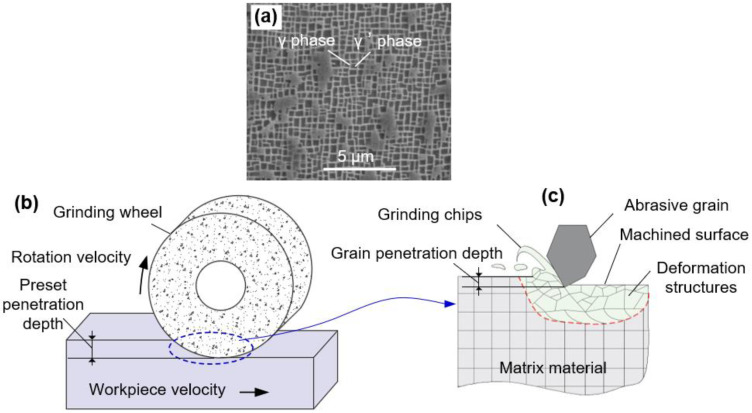
(**a**) The original microstructure of nickel-based superalloy, and schematic illustration of (**b**) the surface mechanical creep-feed grinding process and (**c**) the plastic deformation induced by SMCGT.

The workpiece material used in this study was the single crystal nickel-based superalloy (Beijing Institute of Aeronautical Materials, Beijing, China), which is typically composed of two phases, i.e., γ channels and γ’ particulates, as shown in Figure 1a. The chemical compositions are shown in Table 1. The workpiece was firstly cut to a size of 32 mm × 15 mm × 8 mm (length × width × height), and it was then processed by SMCGT with a water-based emulsion coolant, delivered at a high pressure and high flow rate. As presented in Figure 1b, the workpiece moved in the heroization direction at a velocity V_w_, while the grinding wheel rotated at a velocity Vs with the preset penetration depth Ap. In the grinding process, when one abrasive grit made contact with the workpiece material, plastic deformation occurred, which can result in material pileup and grinding chips (Figure 1c) [22]. Due to the motion and interaction of thousands of abrasive grits in one grinding wheel, the majority of the pileup can be compressed and substantially form the machined surface and the deformed layer. When the wheel advanced to the end of workpiece, the obtained surface was treated by one grinding pass. In the present experiment, a total of three grinding passes were used to increase the material plastic strain and form the final SMCGT sample. A detailed arrangement of the processing parameters can be found in Table 2.

The grinding forces were measured by a three-component platform dynamometer (HR-FP3407 M&T Horizon, Shanghai, China) fixed to the machine bed, and the grinding temperature was tested by using a constantan wire workpiece semi-natural thermocouples. Detail information on the measurements of force and temperature during the grinding process can be found in [23,24]. To observe the microstructure of the original material and the treated surface topography, the scanning electron microscope (S3400, Hitachi, Japan) was employed at a voltage of 20 kV. The elemental distribution in the surface layer was examined using an energy dispersive X-ray spectroscope (EDS) (Xplore Compact 30, Oxford, UK) in a scanning electron microscope. The cross-sectional microstructure of the SMCGT sample was characterized by means of an FEI Tecnai FP 5026 transmission electron microscope. Furthermore, hardness across the sample cross-section was evaluated by a commercial Agilent G200 nano-indenter with a Berkovich diamond tip using the continuous-stiffness measurement technique with a strain rate of 0.05 s^−1^, a loading depth of 100 nm, and an oscillation force frequency of 45 Hz. In particular, the nanoindentations at the same depth were repeated six times, and the results were averaged.

## 3. Results

### 3.1. SEM Characterization of Surface Layer

**Figure 2:**
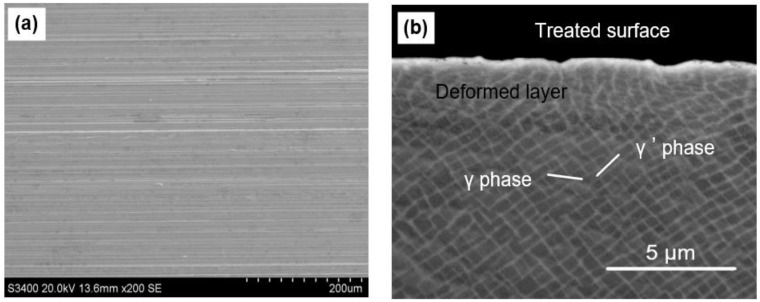
(**a**) Treated surface and (**b**) cross-sectional microstructure of SMCGT sample.

Figure 2a shows the treated surface morphology of the SX alloy after SMCGT. It can be seen that the processed surface is smooth without observable defects, except for some slight material pileups that appear on the sides of abrasive grain motion traces due to the plastic flow of the material. Figure 2b presents the cross-sectional microstructure of the SMCGT sample. As can be seen from Figure 2b, plastic deformation in the surface layer of the SX alloy is obvious and exceeds a depth of around 4 µm. In addition, the bending level of the original structures, i.e., the grids composed of γ and γ’ phases with a size of about 600 nm, increases, evidently approaching the treated surface. Furthermore, in the top 1 µm, the original structures could hardly be identified by the current SEM. No crack was observed in the deformed surface layer of the SMCGT sample.

### 3.2. TEM Characterization of Surface Layer

#### 3.2.1. Cross-Sectional Structures

**Figure 3:**
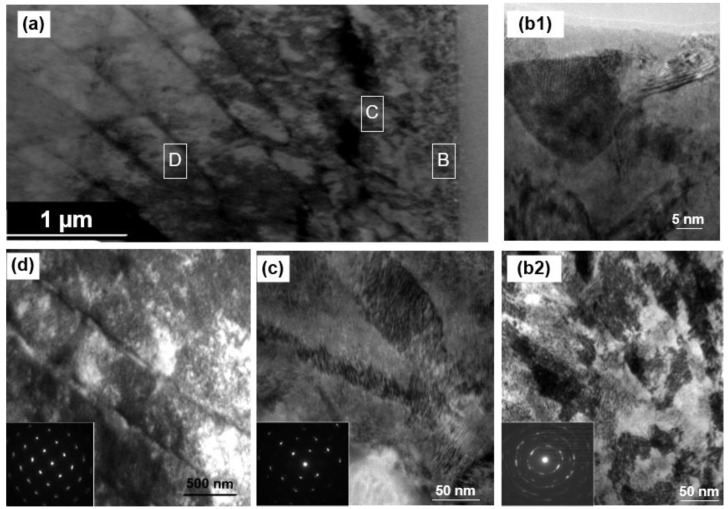
(**a**) TEM cross-sectional observation of the SMCGT sample; (**b–d**) TEM observations corresponding to the marked regions in (**a**): (**b1**) and (**b2**) in region B (0 to 0.5 µm deep from the surface), (**c**) in region C (1 µm deep from the surface), and (**d**) in regions D (2 to 3 µm deep from the surface).

As shown in Figure 3a, the overall distribution of the typical nano–microstructure along the depth of the surface layer could be observed. In the top 0.5 µm thick layer (region B marked in Figure 3a), there exists extensive nano-sized grains (Figure 3(b1,b2)). The selected area electron diffraction (SAED) pattern inserted in Figure 3(b2) suggests that the crystal orientations of these nano-sized grains are random. In the depth range of 0.5–1 µm, lamellar structures of sub-micron sizes are produced in region C, as shown in Figure 3c. The change in contrast within the lamellae is clear due to the difference in orientation gradients induced by a variety of crystalline defects [25]. The rotating SAED pattern in Figure 3c indicates that it is probably a mixing structure of a single crystal and polycrystal. This could be the transition layer, which is actually between the two types of microstructures. Moreover, various dislocation structures can be found in the depth range of more than 2 µm, as demonstrated in Figure 3d with a slightly disturbed SAED pattern. This suggests that the pronounced dislocation activities are created in region D during SMCGT.

**Figure 4:**
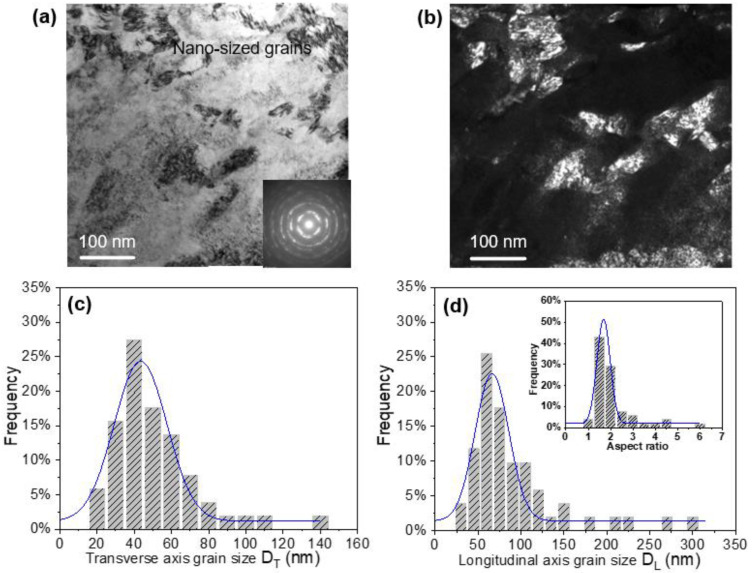
TEM observations and statistical distribution of grain size in the top surface layer of SMCGT sample: (**a**) bright field image; (**b**) dark field image; (**c**) transverse axis grain size (D_T_); and (**d**) longitudinal axis grain size (D_L_) (inset: aspect ratio (D_L_/D_T_) distribution).

In order to obtain further information on grain size distribution in the top surface layer of the SMCGT sample, the grains within the top surface layer (0 to 0.5 µm deep from the surface) were counted through TEM characterization, which is shown in Figure 4 by a histogram with a normal logarithmic distribution. As displayed in Figure 4a,b, both the bright and dark field images present the nano-sized grains in the top surface layer. The nano-grains, which have random crystallographic orientations, can be indicated by the inserted SAED pattern (Figure 4a). It can be also found that that the transverse axis grain sizes (D_T_) vary from 20 to 135 nm (in average 43 nm, Figure 4c), and the longitudinal axis grain sizes (D_L_) range from 30 to 315 nm (averagely 81 nm, Figure 4d). The small value of the aspect ratio (about 1.8) of the nano-sized grains can be indicative of approximately equiaxed grains. Ding et al. [13] observed nanostructures with a size of 20–70 nm in the surface layer of a C-22HS nickel-based alloy after SMCGT, and Torre et al. [26] also obtained refined crystalline grains (around 105 nm in size on average) in a Ni alloy through HPT. These results are comparable to that shown in Figure 4. Therefore, from both Figure 3 and Figure 4, it can be stated that a gradient nano–microstructure is achieved with a generally increasing grain size from the nano scale to the sub-micron and micron scales in the surface layer of the SX alloy after SMCGT.

#### 3.2.2. Dislocation Movement

The above analysis demonstrates that strong plastic deformation in the superficial layer refines the microstructure of the SX alloy into micro-sized laminates and nano-sized grains. Further, the formation of these refined structures mainly results from the dislocation movement at a nano scale induced by high strain, strain rate, and strain gradient in the process of SMCGT [14]. In the current study, due to the decreasing plastic deformation along the depth from the treated surface to the matrix material, significant gradient changes in strain and strain rate were achieved. Thus, the dislocation movements in the surface layer are different along the depth, and they can be characterized via observations of the dislocation morphology of the SX alloy.

**Figure 5:**
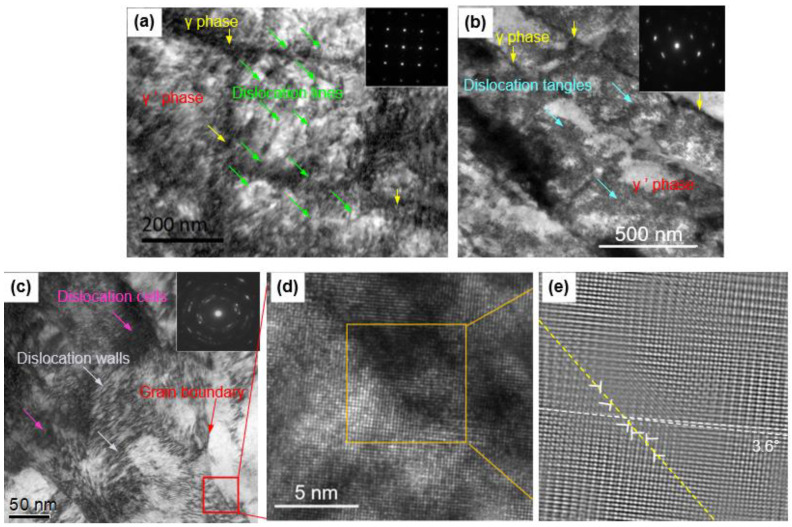
Typical dislocation morphology in SX alloy at different plastic deformation levels: (**a**) low; (**b**) middle; and (**c**) high; and (**d**) high-resolution TEM image of the red boxed area in (**c**). (**e**) The Fourier-filtered image corresponding to the yellow area in (**d**).

In Figure 5a, at a low level of plastic deformation, it can be found that a large amount of dislocation lines is produced within the original γ’ precipitates. Particularly, many dislocation lines also pass through the γ channels. This indicates that the dislocation movement occurs not only inside the γ’ phases, but also within the γ phases. Moreover, the majority of dislocation lines run through the entire grids in a direction that is parallel to the workpiece moving velocity. At this moment, the γ channels and γ’ precipitates are relatively distinct and have clear boundaries, as presented in Figure 5a, though they are plastically deformed and are coupled with dislocations. The SADE pattern showing a sharp and orderly single crystal lattice implies that there is no crystallite rotation or crystallite generated in this layer. However, with the increasing strain and strain rate (Figure 5b), dislocation multiplications are accelerated, developing into dense dislocation tangles in the SX alloy. The boundaries between the two types of primary phases become indistinct and even disappear. This is an actual transition process that offers the opportunity for high-density dislocations to further annihilate and rearrange subdividing sub-micron grains or laminate when, at a level of high plastic deformation (Figure 5c), neither γ nor γ’ phases can be observed. According to research [27,28], the strain, strain rate, and strain gradient during SMCGT can be assessed roughly as high as 6–8, 10^6^–10^7^ s^−1^ and 0.8–1.5 µm^−1^, respectively, based on the Merchant theory in metal-cutting mechanics [29]. Under such conditions, high-density dislocation walls are caused and further develop into dislocation cells with a size of 20–50 nm (Figure 5c). Dense dislocation structures could generate a network owing to the accommodation of dislocations in different orientations under strong shear deformation [30]. Additionally, the structures of lamellar boundaries and sub-grains can be formed if substantial dislocations are induced progressively and are incorporated into dislocation cell boundaries. One of the grain boundaries boxed by a red line in Figure 5c is magnified in a high-resolution TEM image (Figure 5d). The further Fourier-filtered image (Figure 5e) presents the atomic lattice structures of the two newly formed grains across the boundary (marked by a yellow dashed line), and shows the low angle boundary with a misorientation angle of about 3.6° (marked by a white dashed line). The small changes in crystal orientation suggest that it might be suppressed for the dislocation boundaries developed from a low misorientation angle to high ones during SMCGT [31]. In addition, the high-density dislocations labeled by ‘T’ symbols shown in Figure 5e are identified typically along the grain boundaries, leading to misorientation across boundaries.

### 3.3. Formation Mechanism of Gradient Structures

Structural refinement is highly dependent on dislocation activities, which are dominated by plastic deformation parameters, i.e., deformation mode, strain, strain rate, strain gradient, and deformation temperature [32]. In the present SMCGT process, the matrix materials are deformed because of the mechanical and thermal effects resulting from the strong interaction between high-speed rotating abrasive grits and the workpiece material. Furthermore, the mechanical effect causes high material plastic strain, strain rate, and strain gradient, facilitating the dislocation generation and resulting in structural refining and work-hardening, while the thermal effect induces the large deformation temperature, promoting the dynamic recovery of dislocations and leading to structural coarsening and softening.

**Figure 6:**
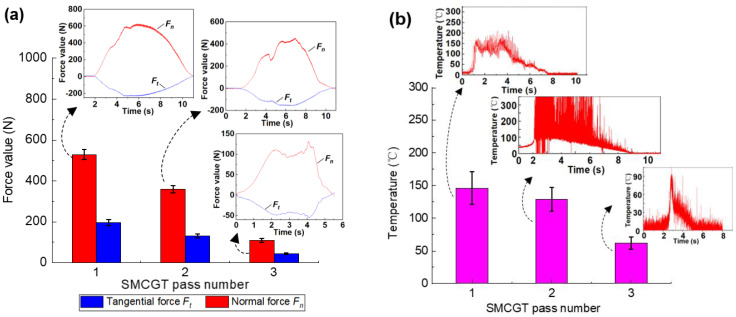
(**a**) The grinding forces and (**b**) grinding temperatures during SMCGT.

Therefore, to clarify the competitive predominance between the mechanical and thermal effects influencing plastic deformation parameters is of great importance in analyzing the formation mechanism of gradient structures. Thus, both force and temperature during SMCGT were measured as shown in Figure 6. It can be observed in Figure 6a that the normal forces *F_n_* (i.e., approximately 529 N for SMCGT pass No.1, 360 N for SMCGT pass No.2, and 100 N for SMCGT pass No.3, respectively) are almost two to three times bigger than the corresponding tangential forces *F_t_* (i.e., 196, 131, and 44 N, respectively). Generally, the normal force is to press abrasive grits into the workpiece material and the tangential force is to form the material pileup and grinding chips [33]. In other words, the workpiece material has to endure large pressure during SMCGT while generating severe plastic deformation. Interestingly, the temperature values during SMCGT are very low, (i.e., 146 °C for SMCGT pass No.1, 129 °C for SMCGT pass No.1, and 62 °C for SMCGT pass No.1, as shown in Figure 6b), without any burnout of the workpiece. This means that, due to the unique conditions used in the grinding process (i.e., the slow workpiece moving velocity, relatively large preset penetration depth, and high-pressure cooling), most of the grinding heat could be carried away, thus yielding the low temperature of the machined surface. This is supported by extensive previous studies on the grinding characteristics of the creep-feed grinding process [33,34].

To further evaluate the mechanical and thermal effects on structural refinement, the Zener–Hollomon parameter *Z* is introduced as follows [35]:

(1) $$Z=\dot{\varepsilon }\;\exp\left(\frac{Q}{RT}\right)$$

where *R* is the constant of gas, $\dot{\varepsilon }$ is the strain rate, *Q* is the activation energy, and *T* is the deformation temperature. According to Equation (1), it can be found that if the strain rate is increased and the deformation temperature is reduced, the effectiveness of structural refining can be improved. In the case of the mechanical effect, due to the very high grinding force (e.g., 529 N, as shown in Figure 6a) and high wheel-rotation velocity, the strain and strain rate in current SMCGT conditions can reach 5–7 and 10^6^–10^7^ s^−1^, respectively. With the high strain rate, there is no sufficient time for dislocations to activate dynamic recovery. On the other hand, as shown in Figure 6b, the grinding temperatures of less than 150 °C are much lower than that of the melting point of the SX alloy (~1342 °C). At such a low grinding temperature, the thermal-induced structural coarsening would be suppressed significantly due to the high speed of the moving heat source (i.e., the abrasive grit engagement with the workpiece material) and the small thickness of the thermal affected zone (Figure 2b) [36]. The mechanical effect becomes more pronounced than the thermal effect. Under such circumstances, more system energy is expected to be stored in the form of crystalline defects (e.g., dislocation lines, tangles, and walls); thus, the enhancement of dislocation density can be facilitated according to the increasing Zener–Hollomon parameter Z (Equation (1)).

**Figure 7:**
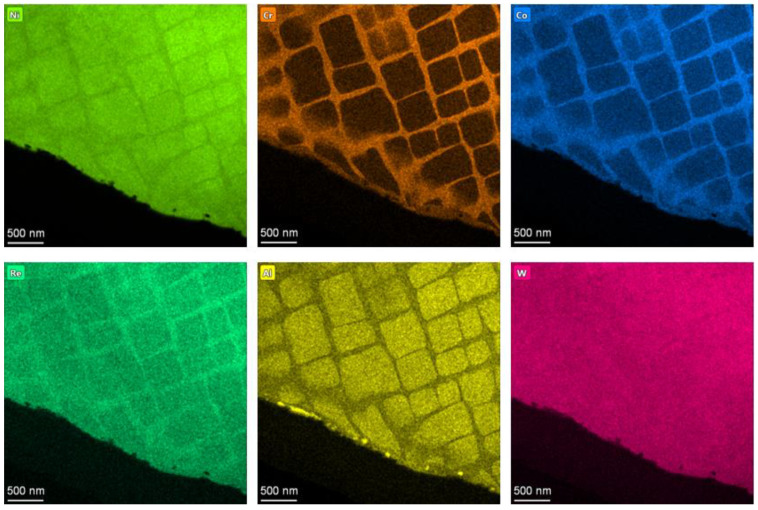
EDS maps of elemental distribution in the surface layer of SX alloy.

In addition, as indicated by Figure 7 which shows the EDS maps of elemental distribution in the surface layer of the SX alloy after SMCGT, the atomic diffusion of Cr and Co within the γ channels can be found. It seems to be very slight, although the plastic deformation of the γ and γ’ phases is apparent. Thus, it can be stated that, owing to the very low temperature and very short time duration during SMCGT, atomic diffusions between the constitutive elements within the γ and γ’ phases would be frozen. A similar phenomenon was observed in the top surface layer of a workpiece by Liao et al. [25] who reported the grain refinement mechanism of a polycrystalline nickel-based superalloy by mechanical cutting. Consequently, in the present study, the structural refinement of the SX alloy can be attributed to the dominantly mechanical effects during SMCGT.

It is worth mentioning that when the strain gradient is introduced, geometrically necessary dislocations (GNDs) are required to maintain the compatibility of deformation. Theoretically, GND density has a linear relationship with the strain gradient as follows [37]:

(2) $$\rho_{G}=\frac{4\chi }{\sqrt{3}b}$$

where *ρ_G_* is the GND density, *χ* is the strain gradient, and *b* is the Burgers vector (0.33 nm for Ni [38]). For the present study, because of the unique depth-dependent contact process between the abrasive grit and workpiece material, the variation of stain is inhomogeneous, inevitably resulting in a large strain gradient in the surface layer. According to Equation (2), a strain gradient of 0.8–1.5 µm^−1^ corresponds to a GND density of 5.6–10.5 × 10^15^ m^−2^. This means that the GND density is so high that more crystalline defects have to be created to store dislocations and provide a compatible deformation. Meanwhile, in this process, the interactions within these high-density dislocations are believed to subdivide the microstructures in turn, subsequently leading to the refinement of grains. Previous reports have demonstrated that the strain gradient can effectively incorporate extreme refined structures into Ni [27], Al [31], and Cu [39]; therefore, it becomes one of the critical parameters to promote structural refinement, although the strain gradient values induced by surface treatments are different. This suggests that by quantitatively controlling the strain gradient and strain rate, the acceptable refined structures, together with outstanding mechanical properties, could be achievable if proper mechanical surface treatments are chosen.

**Figure 8:**
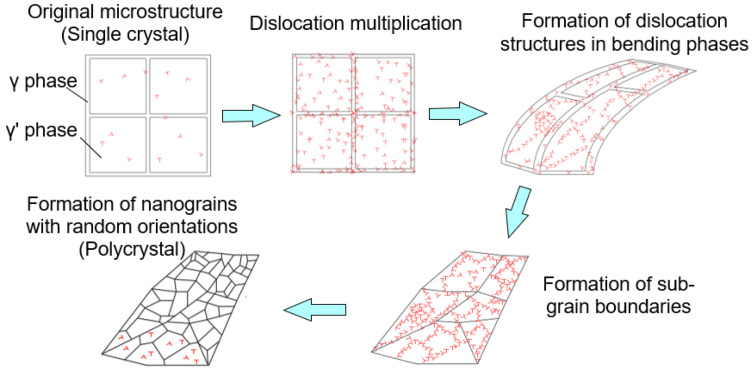
Schematic illustration of the gradient structure evolution.

The results of the above analysis regarding the evolution of gradient structures in the SX alloy under SMCGT can be summarized as displayed in Figure 8. Firstly, high-density dislocations are created due to the large strain and strain rate induced by strong interactions between abrasive grits and workpiece materials. Then, with the increasing strain and strain rate, the grids composed of γ and γ’ phases start to bend at a macro scale. At the same time, the strain gradient is formed, inducing a great elevated density of GND within the γ and γ’ phases. These dislocations develop into various dislocation structures, i.e., dislocation tangles and walls, and the generation of more dislocation boundaries is facilitated in order to accommodate high-density GNDs. Thirdly, the dislocation boundaries might transform into low-angle sub-micron-sized grains or lamellar structure boundaries by progressively absorbing the accumulated dislocations. Finally, when the strain, strain rate, and strain gradient approach the highest values, the sub-micron-sized grains and lamellar structures become finer and finer, eventually forming nano-sized grains.

### 3.4. Effect of Gradient Structures on Hardness

**Figure 9:**
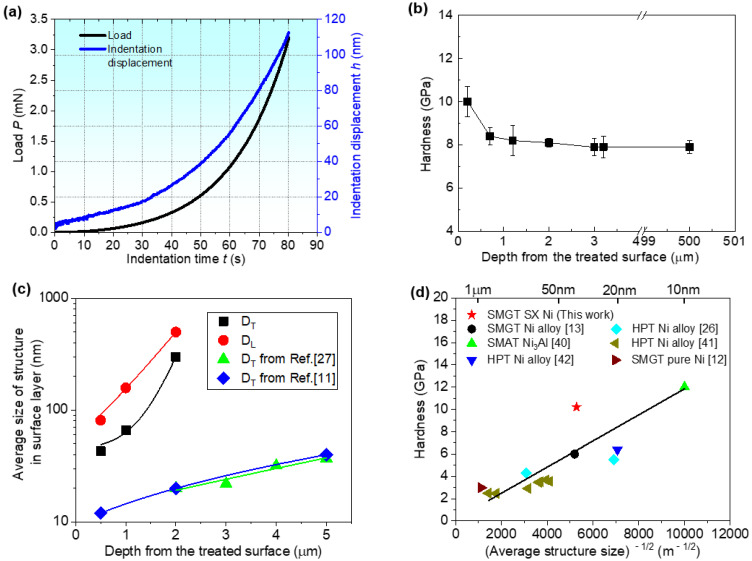
(**a**) Typical curves of load and displacement with time, and variations of (**b**) hardness and (**c**) structure size along depth from the treated surface, and (**d**) hardness vs. structure size for the SMCGT sample.

With increasing depth from the SMCGT surface, the grain size of the SX alloy in the surface layer increases, and the corresponding hardness follows the principle of ‘smaller is stronger’ [14]. Figure 9 shows the results of hardness of nanoindentation. The typical curves of load and displacement with time can be seen in Figure 9a. It can be found from Figure 9b,c that, the gradient structure corresponds to a gradient distribution of hardness with increasing depth. That is, the nanoindent closer to the topmost surface presents a larger value of hardness. When grain size reaches the minimum value (around 43 nm), a maximum hardness of about 10.2 GPa is obtained (Figure 9b). Both the structure-size-depth profile and the hardness-depth profile are similar to those reported for the structurally gradient C-22HS nickel-based alloy by SMAT [13] and pure nickel by SMGT [12]. As shown in Figure 9d, the detail data collected from [11,12,13,26,27,40,41,42] generally follow the empirical Hall–Petch relation, which presents an increasing trend in hardness with the reciprocal square root of the grain size. It can be also found from [13] that the structural sizes of the C-22HS nickel-based alloy and pure nickel can be refined to 21 and 12 nm, corresponding to the hardness values of 5.5 and 12 GPa, respectively. However, for the present sample of the SX alloy, a hardness of about 10 GPa may be the highest if the structure size is identical. This value correlates with the structures of nano-sized grains. Furthermore, hardness values of 8.1 and 7.9 GPa correspond to the micro-sized lamellar structures and matrix materials of the SX alloy, respectively (Figure 9b,c). In addition, Figure 9d shows that the above experimental hardness values sit above the Hall–Petch line in the current study. This might be attributed to the coupled effects from the essential properties strengthened by various rare earth elements, (e.g., Re [20]), and from the grain refinement by SMCGT in the surface layer. Thus, it is demonstrated that incorporating the gradient nano–micro-sized structures into the surface layer of the SX alloy by means of SMCGT is expected to further enhance the overall mechanical properties and even fatigue properties, and further exploration is therefore deserved.

## 4. Conclusions

(1) Gradient structures (i.e., nano-sized grains, sub-micron-sized grains, and lamellar structures, and dislocation structures) were created in the machined surface layer of a single crystal nickel-based superalloy during the surface mechanical creep-feed grinding treatment process.

(2) The formation of gradient structures was governed by dislocation movements that run through both γ channels and γ’ precipitates thoroughly, subdividing the original structure of the single crystal into various dislocation structures, eventually leading to grain refinement in the surface layer.

(3) The creep-feed grinding process produced a large grinding force (up to 529 N) with a low grinding temperature (less than 150 °C) in the present study, substantially resulting in the graded variation of strain and strain rate, under which the accommodated GNDs were enhanced while the dynamic recoveries were suppressed. Thus, the evolution of gradient structures mainly originates from the mechanical effect between abrasive grits and the workpiece material during SMCGT.

(4) In the top surface layer of the SX alloy, the hardness of nanostructures has the largest value of about 10 GPa, which is around 26% higher than that of matrix material.

Because the nickel-based superalloys are commonly in the aero-engine industry, if gradient structures can be introduced into the surface layer, this might have beneficial effects on the enhancement of the service performance (such as expending fatigue life) of critical components of an aero-engine; therefore, this deserves in-depth investigation in the future.

## Figures and Tables

**Table 1:** Chemical compositions of the nickel-based superalloy used in study [20].

Elements	C	Cr	Co	Mo	Al	Ti	Fe	W	Ta	Re	Nb	Hf	Ni
Contents/wt.%	0.01	4.3	9	2	5.7	0.05	0.21	8	7.5	2	0.5	0.1	Bal.

**Table 2:** Processing parameters during SMCGT.

Grinding Pass Number	Grinding Wheel-Rotation Velocity Vs (m/s)	Workpiece Moving Velocity Vw (mm/min)	Preset Penetration Depth Ap (mm)
1	25	240	1.50
2	35	210	1.00
3	35	480	0.13

## Data Availability

The data presented in this study are available on request from the corresponding author.
